
JOURNAL INFORMATION
==============================
NLM Title Abbreviation: J Vet Intern Med
Journal ID: J Vet Intern Med
NLM Journal ID: 8708660
Journal ID: jvim

Journal of Veterinary Internal Medicine

ISSN: 0891-6640
EISSN: 1939-1676
Publisher: Oxford University Press

ARTICLE INFORMATION
==============================
PMCID: PMC13505507
DOI: 10.1093/jvimsj/aalag185
Article ID: aalag185
Article version: 1
Subjects: Abstracts, AcademicSubjects/SCI01400

2026 ACVIM Forum Research Report Program June 10 – October 31, 2026

Publication date: 2026 Jul-Aug
Electronic publication date: 2026 Aug 25
Volume: 40
Issue: 4
Electronic Location ID: aalag185
Received 2026 Jul 24; Accepted 2026 Jul 24
Copyright: © The Author(s) 2026. Published by Oxford University Press on behalf of the American College of Veterinary Internal Medicine.
Copyright year: 2026
License: This is an Open Access article distributed under the terms of the Creative Commons Attribution License (https://creativecommons.org/licenses/by/4.0/), which permits unrestricted reuse, distribution, and reproduction in any medium, provided the original work is properly cited.
License URL: https://creativecommons.org/licenses/by/4.0/

Page count: 13

==============================
The American College of Veterinary Internal Medicine (ACVIM) Forum and the Journal of Veterinary Internal Medicine (JVIM) are not responsible for the content or dosage recommendations in the abstracts. The abstracts are not peer reviewed before publication. The opinions expressed in the abstracts are those of the author(s) and may not represent the views or position of the ACVIM. The authors are solely responsible for the content of the abstracts.

